# A Case of Atypical Multiple Sclerosis Mimicking Cerebral Autosomal Dominant Arteriopathy With Subcortical Infarcts and Leukoencephalopathy Syndrome

**DOI:** 10.7759/cureus.12508

**Published:** 2021-01-05

**Authors:** Asuman Orhan Varoğlu, Begumhan Baysal, Umit Goren

**Affiliations:** 1 Neurology, Istanbul Medeniyet University, Istanbul, TUR; 2 Radiology, Istanbul Medeniyet University, Istanbul, TUR

**Keywords:** multiple sclerosis (ms), subcortical ischemic leukoencephalopathy (cadasil), atypical demyelinating lesions

## Abstract

It remains important to be able to distinguish between multiple sclerosis (MS) and cerebral autosomal dominant arteriopathy and subcortical ischemic leukoencephalopathy (CADASIL), although it has yet to be reported that MS and CADASIL can be seen together.

We encountered a 63-year-old female patient compatible with MS in terms of clinical features but compatible with CADASIL in terms of brain magnetic resonance imaging (MRI) findings. Migraine, vascular dementia, and subcortical stroke events, which are among the classic clinical features of CADASIL, were not present. In the cerebrospinal fluid (CSF) examination, the oligoclonal band (OCB) was positive and the NOTCH 3 mutation was negative in the serum. The patient, whose initial symptom was optic neuritis, recovered with IV corticosteroids and azathioprine therapy. The patient's daughter and aunt had previously been diagnosed with MS.

We present a case of MS mimicking CADASIL in terms of atypical demyelinating lesions.

## Introduction

Multiple sclerosis (MS) is an autoimmune demyelinating disease of the central nervous system that results in myelin loss and axonal damage [1]. Cerebral autosomal dominant arteriopathy and subcortical ischemic leukoencephalopathy (CADASIL) syndrome results from Neurogenic locus notch homolog protein 3 (NOTCH 3) mutations in chromosome 19 [2]. Several case reports in the literature that were initially followed up as MS have been determined to be indicative of CADASIL years later [2-5]. Cases of CADASIL with atypical MRI findings and new NOTCH 3 gene mutations have also been reported [6-9]. Unlike these published reports, we presented a patient with MS who showed MRI findings mimicking CADASIL but fulfilled McDonald's clinical criteria for MS [1] and responded well to anti-inflammatory therapy.

## Case presentation

**Figure 1:**
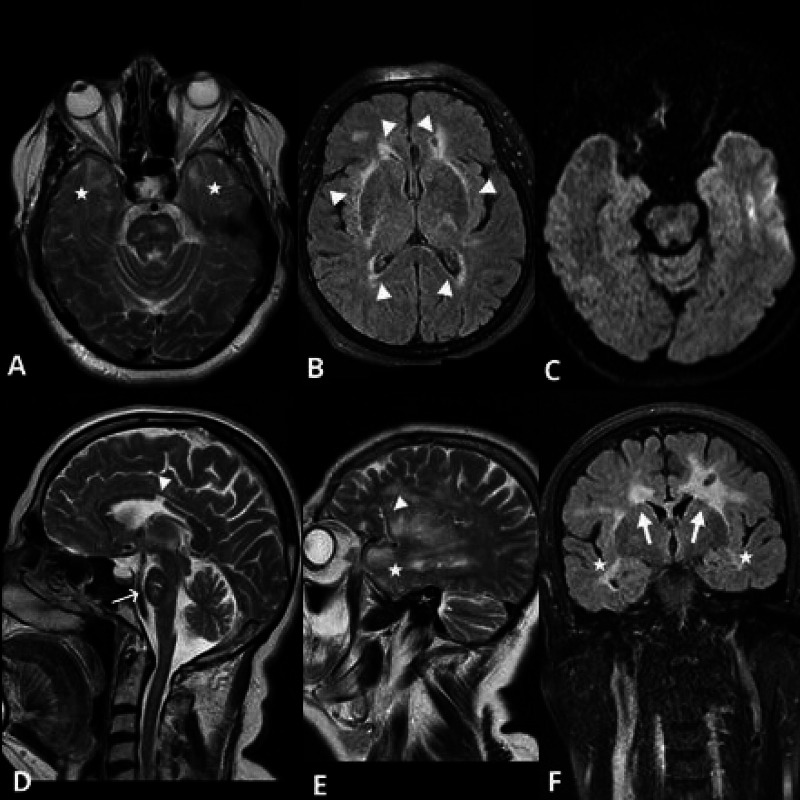
Axial T2 weighted and fluid-attenuated inversion recovery (FLAIR) images Axial T2 weighted and FLAIR images show high signal intensities on bilateral temporal subcortical white matter (asterisks, images A and C). On image B, involvement of the external capsules, posterior limbs of the internal capsules and periventricular white matter (arrow heads) are demonstrated. On sagittal T2 weighted image (image D), we see corpus callosum lesion (arrow head), pontine lesion (thin arrow), and frontal juxtacortical lesions. Parasagittal T2 weighted image (image E) depicts frontal subcortical white matter involvement (thin arrow). There is band-like external capsule involvement (thick arrow) and frontal-temporal (asterisks) periventricular and subcortical white matter lesions appreciated on coronal FLAIR image (image F).

A 63-year-old woman was admitted to our clinic 25 years ago due to weakness in her right arm and leg, speech disorder, and diplopia, which lasted for five days. The neurological examination revealed right-sided hemiparesis, dysarthria, hypoesthesia on the right extremities, and the presence of paresis in the left third cranial nerve. Fundal examination was normal. Axial T2 weighted and fluid-attenuated inversion recovery (FLAIR) images show high signal intensities on bilateral temporal subcortical white matter (Figure 1). She was a non-smoker. The markers of thrombophilia and vasculitis were normal. The cerebrospinal fluid (CSF) analysis was normal. There were OCBs present in the CSF (unmatched in the serum). Therefore, according to modified McDonald's criteria [1], the patient was diagnosed with MS and treated with 50 mg of azathioprine, as she she rejected other treatment. After two years of azathioprine treatment, the patient was hospitalized due to an attack of paraparesis. Azathioprine treatment was continued. In our outpatient clinic, we learned that the patient was treated with psychiatry for depression due to divorce. To treat her depression, she used 20 mg of escitalopram daily for a single year. The patient had no other psychological diseases. We stopped the azathioprine treatment because she had used it for a long time. She was treated with IV methylprednisolone for all her attacks, and improvement was observed. We could not start any treatment because our patient, who was 63 years old, had rejected other treatment for MS. The patient’s Mini-Mental-Status was determined to be 26. Her Expanded Disability Scale Score (EDSS) was 3.5. No mutation was detected in the patient's NOTCH 3 gene's exons 3, 4, 5, and 6. In addition, the patient's daughter and aunt had previously been diagnosed with MS. 

**Figure 2:**
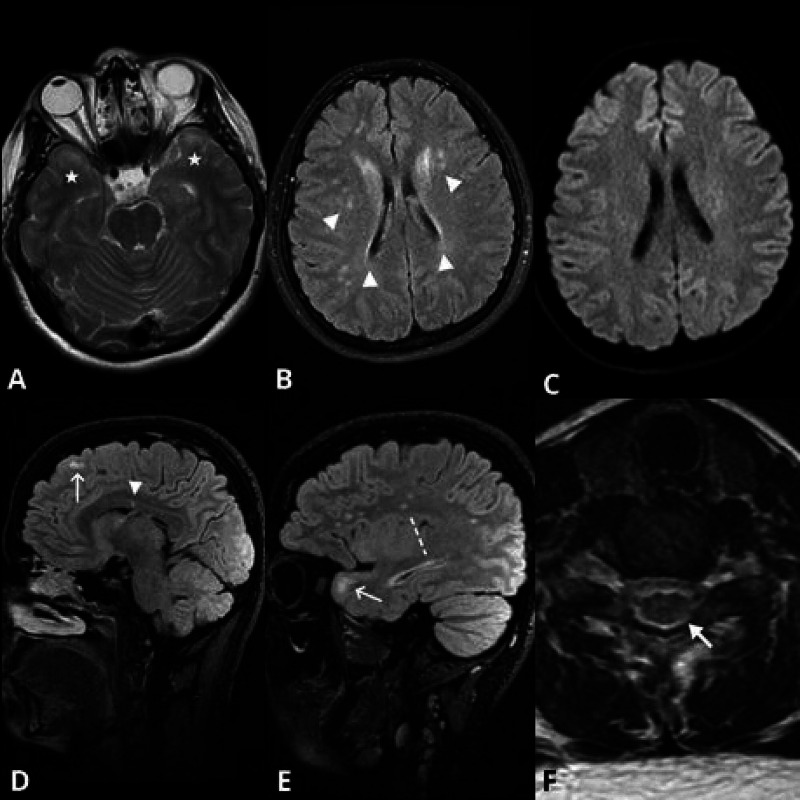
On axial T2 weighted MR and Sagittal 3D CUBE fluid-attenuated inversion recovery (FLAIR) images On axial T2 weighted MR image, increased signal intensity is seen at bilateral anterior temporal subcortical white matter (asterisks, image A). Image B demonstrates periventricular plaques and numerous high signal intensity foci at corona radiata (arrow heads). There is no diffusion restriction appreciated in our case (image C). Sagittal 3D CUBE FLAIR images show corpus callosum lesion (arrow head) and juxtacortical lesions (thin arrows, images D and E). Also, frontoparietal white matter lesions (dashed line) are well demonstrated on image E. In addition to cranial images, on axial T2 weighted image of the spine (image F), there is an increased signal focus (thick arrow) detected at left half of posterior spinal cord.

For the patient’s daughter, we showed that the NOTCH 3 genetic screening was negative as well. When the patient's daughter was 25 years old, she applied to the neurology outpatient clinic due to numbness in her left face, paraparesis, and optic neuritis. OCBs were absent in the CSF investigation, and bilateral visual evoked potentials (VEPs) were long. She used glatiramer acetate for four years to treat MS. At her own request, she stopped using this medicine seven months ago. Later, the patient suffered a left hemiparesis attack and was treated with IV pulse methylprednisolone. Demyelinating lesions compatible with MS were observed in brain and cervical MR (Figure 2). 

The patient and her daughter had presented with no migraine, ischemic events, behavioral disorders, or cognitive disorders. In retinal examination, the retinal cotton wool spot was not observed in either.

## Discussion

CADASIL initially begins with migraine with aura in the second decade, subcortical events in the fourth decade, and vascular dementia late in life. The NOTCH 3 gene mutation is 100% sensitive and specific in cases of CADASIL [10]. This mutation could not be demonstrated in the patient and her daughter. In a few reports, weak antinuclear antibody (ANA) positivity has been reported among vasculitis markers in CADASIL. It has also been reported that CADASIL can be accompanied by other collagen tissue diseases [9,10]. Vasculitis markers were not observed in either of the current report’s cases.

Existing literature has reported a number of cases of CADASIL that have been followed up and misdiagnosed as MS [3-5]. Although it has been reported that the coexistence of MS and CADASIL is rare [9], other authors have asserted that CADASIL and MS cannot co-occur [11]. We were unable to find a published case that identified the coexistence of both MS and CADASIL.

The presence of the OCB is important to the diagnosis of MS. While it is not a pathognomonic sign, it does present in 95% of MS cases [11]. In the current case, CSF investigation revealed OCB but not in her daughter. Schiess reported [8] that a patient who has OCB (unmatched in serum) and cognitive symptoms and incompatible with CADASIL in brain-spine MRI lesion treated with IV steroids and glatiramer acetate. The authors described this condition as MS or “inflammatory CADASIL” [8]. Another author presented a patient with CADASIL who had OCB (unmatched in serum) and spine lesions, which are indicative inflammation. These findings are atypical for CADASIL. This patient was also treated with IV steroids [8]. Optic neuritis can serve as an important indicator of inflammatory processes that are taking place in the background. Optic neuritis was present in our patient and her daughter. In addition, the daughter’s bilateral VEPs were long. Collongeus et al. reported on a patient with an initial presentation of optic neuritis and who was treated with IV corticosteroids and glatiramer acetate [12]. At the time of follow-up, the patient has developed cognitive disorders that were indicative of CADASIL. Our case and her daughter also responded to anti-inflammatory treatment.

Although existing literature has presented information about CADASIL, which has atypical MRI findings, MRI findings for CADASIL are significantly characteristic [6-9]. Atypical MRI findings for CADASIL include contrast enhancement and spinal lesions, which are suggestive of inflammatory diseases. The patients in studies with these findings improved with anti-inflammatory therapy. In all published atypical CADASIL cases, cognitive symptoms that support CADASIL have developed over time. Atypical NOTCH 3 gene mutations have also been demonstrated in patients with atypical MRI readings of CADASIL, and in these cases, recovery has been observed with the administration of anti-inflammatory therapy [6-9]. We believe that these findings are incapable of explaining CADASIL, as atypical MRI findings that were indicative of inflammation and used treatments of anti-inflammatory therapy were also present in these cases. Schiess et al. [8] described this table as “inflammatory CADASIL.” As such, the detection of new and different NOTCH 3 gene mutations is not necessarily related to CADASIL.

Although the present case’s MRI findings showed evidence that was suggestive of CADASIL, the clinical features of this syndrome were not found in either the patient or her daughter. In addition, while NOTCH 3 gene mutations were not detected in either subject, improvements were achieved using IV steroids and glatiramer acetate treatments. Granular osmiophilic material (GOM) examination was not performed, since CADASIL syndrome was not considered because the patient had no family history, no typical clinical findings, and no classic NOTCH 3 gene mutations were found.

Many diseases can be considered in the differential diagnosis of cerebral small vessel diseases. These diseases include Cerebral Autosomal Recessive Arteriopathy with Subcortical Infarcts and Leukoencephalopathy (CARASIL), mitochondrial encephalopathy, lactic acidosis and stroke-like episodes (MELAS) and Cathepsin-A related arteriopathy with strokes and leukoencephalopathy (CARASAL). The most common clinical features of CARASIL are early vascular dementia seizures, psychiatric disturbances, pseudobulbar palsy, spondylosis deformans, seizures and premature head alopecia in 90% of patients. Also, early white matter changes in MRI are located basal ganglia not anterior temporal region. In MELAS, eyelid ptosis, cardiomyopathy, muscle weakness, diabetes and lactic acidosis are seen. Also, characteristic lesions on MRI do not conform to vascular distribution. CARASAL is very rare and mainly involves microangiopathy of retina and brain. Brain lesions can mimic tumors or tumefactive inflammation. We thought that our patient was not compatible with CARASIL, MELAS and CARASAL in terms of both lesions in brain and clinically [13]. For these reasons, we did not consider the CARASIL, CARASAL and MELAS syndromes in our patient.

Despite this, there were findings that suggest CADASIL syndrome as band like external capsule involvement (thick arrow) and frontal- temporal (asterisks) periventricular and subcortical white matter lesions appreciated on coronal FLAIR image in Figure 1F (mother), there were no migraine with aura, ischemic vascular events, cognitive disorders and psychiatric disorders, which are the classic clinical features of CADASIL syndrome.NOTCH3 gene mutation has not been detected and there is no family history for CADASIL syndrome too. Therefore, the mother's diagnosis was not CADASIL. The patient was diagnosed with MS because of the classical clinical features of MS, its progress with attacks and the good response of these attacks to anti-inflammatory (IV steroids) treatment, the presence of oligoclonal bands, and the presence of optic neuritis attacks.

We developed several hypotheses for the current case: (1) although previous literature has not reported the co-existence of MS and CADASIL, these two syndromes may have co-existed; (2) the vascular structure may play an important role in the development of demyelination disorders; (3), there are many variations of CADASIL and MS, some may not even have been described in current literature yet; and (4) the present case is an atypical case of MS mimicking CADASIL in terms of brain lesions.

## Conclusions

In conclusion, we presented a case whose findings were clinically compatible with MS, whose daughter presented with MS, and who responded positively to anti-inflammatory therapy. Nonetheless, a brain MRI scan of the patient showed findings that were similar to CADASIL. It can be said that clinical findings and response to treatment are more valuable than MRI findings in the diagnosis of such patients. We presented a case of MS mimicking CADASIL in terms of brain lesions. We think that this case will shed light on new studies that examine the physiopathology of both CADASIL and MS.
